# A risk model developed based on necroptosis to assess progression for ischemic cardiomyopathy and identify possible therapeutic drugs

**DOI:** 10.3389/fphar.2022.1039857

**Published:** 2022-11-28

**Authors:** Yang Lu, Dashuai Wang, Yaoxi Zhu, Yimei Du, Jinying Zhang, Han Yang

**Affiliations:** ^1^ Department of Cardiology, The First Affiliated Hospital of Zhengzhou University, Zhengzhou, China; ^2^ Department of Cardiovascular Surgery, The First Affiliated Hospital of Zhengzhou University, Zhengzhou, China; ^3^ Department of Cardiovascular Surgery, Zhongnan Hospital of Wuhan University, Wuhan, China; ^4^ Department of Cardiology, Union Hospital, Tongji Medical College, Huazhong University of Science and Technology, Wuhan, China

**Keywords:** necroptosis, ischemic cardiomyopathy, therapeutic drugs, cardiovascular remodeling, signature

## Abstract

**Object:** Ischemic cardiomyopathy (ICM), with high morbidity and mortality, is the most common cause of heart failure. Cardiovascular remodeling secondary to chronic myocardial ischemia is the main cause of its progression. A recently identified type of programmed cell death called necroptosis is crucial in the development of various cardiovascular diseases. However, the function role of necroptosis in cardiac remodeling of ICM has not been elucidated. Our study aimed to screen for genes associated with necroptosis and construct a risk score to assess the progression and evaluate the prognosis of ICM patients, and further to search for potentially therapeutic drugs.

**Methods:** The gene expression profiling was obtained from the GEO database. LASSO regression analysis was used to construct necroptosis-related gene signatures associated with ICM progression and prognosis. TF-gene and miRNA-gene networks were constructed to identify the regulatory targets of potential necroptosis-related signature genes. Pathway alterations in patients with high necroptosis-related score (NRS) were analyzed by GO, KEGG, GSEA analysis, and immune cell infiltration was estimated by ImmuCellAI analysis. CMap analysis was performed to screen potential small molecule compounds targeting patients with high NRS. Independent risk analyses were performed using nomograms.

**Results:** Six necroptosis-related signature genes (STAT4, TNFSF10, CHMP5, CHMP18, JAK1, and CFLAR) were used to define the NRS, with areas under the ROC curves of 0.833, 0.765, and 0.75 for training test, test set, and validation set, respectively. Transcription factors FOXC1 and hsa-miR-124-3p miRNA may be regulators of signature genes. Patients with higher NRS have pathway enriched in fibrosis and metabolism and elevated nTreg cells. AZD-7762 may be an effective drug to improve the prognosis of patients with high NRS. A feature-based nomogram was constructed from which patients could derive clinical benefit.

**Conclusion:** Our results reveal 6 necroptosis gene signatures that can evaluate the progression and prognosis of ICM with high clinical value, and identify potential targets that could help improve cardiovascular remodeling.

## 1 Introduction

Ischemic cardiomyopathy (ICM) is among the most common causes of morbidity and mortality worldwide (Moroni et al., 2021). Ischemic cardiomyopathy is considered a special type of coronary heart disease (CAD) or a consequence of CAD in advanced stages. Its pathological process is long-term myocardial ischemia and hypoxia caused by atherosclerotic lesions (Panza et al., 2021). The severity of ischemic cardiomyopathy is mainly related to the grade of left ventricular (LV) dysfunction and the degree of coronary artery stenosis (Anversa and Sonnenblick, 1990).

Current diagnostic criteria for heart failure (HF) are based on the assessment of left ventricular ejection fraction (EF) which are the standard for grading patients with HF. Importantly, left ventricular systolic dysfunction is an independent predictor of adverse outcomes in patients with HF (Calderon-Dominguez et al., 2021). Increased mortality is strongly associated with reduced left ventricular ejection fraction during both hospitalized and readmitted patients. Randomized clinical trials have shown that severely reduced EF, below 15% at increased absolute risk of death due to arrhythmia and worsening HF (Gajanana et al., 2016). The prognosis of individuals with ICM can be determined with the aid of natriuretic peptides (NPs) and other indicators (Castiglione et al., 2022). However, these results lack specificity as there is a significant variability in NPs levels across the spectrum of HF (Volpe et al., 2016). Highly time-consuming imaging technique limited the assessment of the left ventricular systolic function in cardiac patients with LV dysfunction (Smiseth et al., 2016). Therefore, there is an urgent need to develop new, reliable tools for ICM risk stratification, which can guide the formulation of more effective and individualized therapeutic approaches for these patients.

Necroptosis is a type of non-apoptotic programmed cell death pathway, also called autophagy-induced cell death, which show have play an important role in the immune systems (Khoury et al., 2020; Bertheloot et al., 2021). A growing body of research has shown that necroptosis can trigger inflammation, which can contribute to the development of cardiovascular remodeling in ICM. This suggests that ICM may be diagnosed and treated by focusing on necroptosis (Piamsiri et al., 2021).

In this study, we performed a systematic bioinformatics analysis of ICM patients whose data were deposited in the Gene Expression Omnibus (GEO) database. Screening of necroptosis-related prognostic genes by identifying differentially expressed necroptosis genes and least absolute shrinkage and selection operator (LASSO) regression. We constructed a necroptosis-related score to assess ICM progression and prognosis, and successfully divided ICM patients into two subtypes based on necroptosis-related scores. We explored differences between patients with high and low necroptosis-related score (NRS) in terms of pathway enrichment, immune cell infiltration, and identified targeted drugs for patients with high NRS. A nomogram was constructed to improve risk stratification for ICM patients.

## 2 Materials and methods

### 2.1 Data acquisition

The microarray data and clinical data of the GSE5406 dataset were obtained using the R package “GEO query” (Barrett et al., 2013). GSE5406 (Hannenhalli et al., 2006) containing 108 myocardial tissues from patients with ischemic cardiomyopathy and 16 non-failing donor myocardial tissues. The clinical data was obtained from Andreas et al. (Barth et al., 2011). The GSE57338 and GSE203160 datasets were used to validate the expression of the genes and the accuracy of the model.

### 2.2 Selection of necroptosis-related genes

Differentially expressed genes (DEGs) between ICM and NF were screened using the “limma” package, with an adjusted *p*-value of less than 0.05 as the threshold. Volcano and heat maps of DEGs were generated using the “ggplot2” package. Necroptosis-related genes were collected from the necroptosis pathway (hsa04217) from the Kyoto Encyclopedia of Genes and Genomes (KEGG) database. Necroptosis-related differentially expressed genes (NRDEGs) were identified through an interactive website (www.interactivenn.net).

### 2.3 Screening of key genes

LASSO regression is used to select variables in linear regression by shrinking coefficient values and setting some values to zero to improve prediction accuracy and data interpretation, which can improve data rationality and forecast accuracy. The “Caret” package was used to randomly allocated the samples into training and validation sets with a ratio of 7:3. LASSO regression was processed using the “glmnet” package, setting the observation value of patients with LVEF = 15% to 1 and patients with LVEF >15% to 0, and 21 necroptosis-related genes as independent variables to construct the correlation with LVEF genetic model. Receiver operating characteristic (ROC) curve and area under the curve (AUC) were used to evaluate the predictive effect of the model (Robin et al., 2011). The necroptosis-related score (NRS) was calculated using the following equation: NRS = Σ(βi * Expi) (β: coefficient, Exp: gene expression level). ICM patients were classified into low and high NRS subgroups based on the median NRS.

### 2.4 Functional enrichment analysis

The “limma” package was used to analyse the DEGs in patients with high and low NRS scores. The R package “clusterProfiler” was used to perform gene ontology (GO) and KEGG pathway enrichment analysis of DEGs. In addition, the “clusterProfiler” package was used to perform gene set enrichment analysis (GSEA) on potential mechanisms of c2 (c2. cp.v7.5.1. symbols.gmt) in the Molecular Signature Database (MSigDB) (Yu et al., 2012). Pathways with false discovery rates less than 0.05 were considered statistically significant.

### 2.5 Gene regulatory network analysis

We integrate information derived from two different databases (JASPAR and ChEA) for predicting gene interactions *via* Network Analyst v3.0 network tool (www.networkanalyst.ca). We took the intersections of TF-gene interactions obtained from JASPAR and ChEA. We used the miRTarBase, a database holding knowledge of miRNA targets from different organisms, to predict miRNA-gene interaction. The Cystoscope’s Cryotube plugin (Shannon et al., 2003) was used to provided topological analysis of this network and connectivity and retain the top 30 nodes of connectivity for further analysis.

### 2.6 Immune infiltration analyses

The Immune Cell Abundance Identifier (ImmuCellAI, bioinfo. life.hust.edu.cn/ImmuCellAI) (Miao et al., 2020) was used to calculate the immune cell abundance in myocardial tissue of ICM patients. ImmuCellAI is a gene set signature-based immune cell abundance assessment method for accurate abundance estimation of 24 immune cell types (18 T cell subsets) from gene expression data. To find differences between immune cell infiltration levels between high and low NRS categories, the Wilcoxon rank sum test was performed. To investigate the relationship between important regulators and immune cells, Spearman correlation was used.

### 2.7 The connectivity map (CMap) analyses

The CMap (https://clue.io/) (Musa et al., 2018), a web-based database that exploits differences in gene expression following treatment of human cells with different disruptors to create a database of biological applications where small-molecule compounds, gene expression and disease are interrelated. CMap was used to interrogated the DEGs from patients with high and low NRS, and a correlation score was obtained based on the enrichment of DEGs in the reference gene expression profile. Enrichment scores >90 was considered promising candidate small molecule compounds, and the mode of action analysis (MoA) analysis was performed to predict their mode of action.

### 2.8 Establishment of a nomogram

The “rms” package was used to incorporate NRS and clinical characteristics to create a nomogram. Calibration curves were used to assess the accuracy of the nomogram. The clinical usefulness of the nomogram was assessed by decision curve analysis.

### 2.9 Human samples

Six patients with ICM who had heart transplantation provided left ventricular myocardial tissue. At least 3 months before receiving a heart transplant, all patients had been diagnosed with ICM and an EF of less than 40%. Three organ donors whose hearts could not be transplanted owing to size concerns, ABO mismatches, or other factors were able to provide non-failing (NF) tissue. The study conformed with the Helsinki Declaration (revised 2013). The Ethics Committee of Union Hospital, Tongji Medical College, Huazhong University of Science and Technology evaluated and approved the study (Wuhan, China; approval number: UHCT-IEC-SOP-016-03-01). All patients or their families provided their written informed consent. The clinical data of the 6 ICM patients are shown in Supplementary Figure Table 1.

**TABLE 1:** The sequences of the primers used.

Gene	Forward (5′ →3′)	Reverse (5′ →3′)
STAT4	TGT​TGG​CCC​AAT​GGA​TTG​AAA	GGA​AAC​ACG​ACC​TAA​CTG​TTC​AT
TNFSF10	TGC​GTG​CTG​ATC​GTG​ATC​TTC	GCT​CGT​TGG​TAA​AGT​ACA​CGT​A
CHMP5	AGA​TTT​CTC​GAT​TGG​ATG​CTG​AG	TGT​TGG​GCA​AGA​TTG​TCC​CG
CHMP1B	AAA​GAA​CTG​AGT​AGG​AGT​GCC​A	TGT​ATC​CTC​GCA​ACT​TCC​ATG​T
JAK1	CTT​TGC​CCT​GTA​TGA​CGA​GAA​C	ACC​TCA​TCC​GGT​AGT​GGA​GC
CFLAR	TGC​TCT​TTT​TGT​GCC​GGG​AT	CGA​CAG​ACA​GCT​TAC​CTC​TTT​C
GAPDH	TGT​GGG​CAT​CAA​TGG​ATT​TGG	ACA​CCA​TGT​ATT​CCG​GGT​CAA​T

### 2.10 Real-time polymerase chain reaction (PCR)

Total RNA was extracted from frozen heart tissue using TRIzol reagent (Invitrogen) and PrimeScript RT kit (TaKaRa Biotechnology) was used to reverse transcribe the RNA to cDNA. Real-time fluorescent quantitative PCR was performed using SYBR green (Vazyme). Relative gene expression was calculated using the 2^−ΔΔCT^ method. GAPDH was used as a reference gene. The sequences of the primers are listed in Table 1.

### 2.11 Statistical analysis

All statistical tests were implemented utilizing R software 3.6.1. Data are expressed as mean ± SD. Student’s t-test was utilized for analyzing the difference between the two groups. The correlation between the variables were determined using Spearman’s correlation test. All statistical *p*-values were two-sided, and *p* < 0.05 was regarded as statistical significance.

## 3 Results

### 3.1 Identification of NRDEGs

**FIGURE 1:**
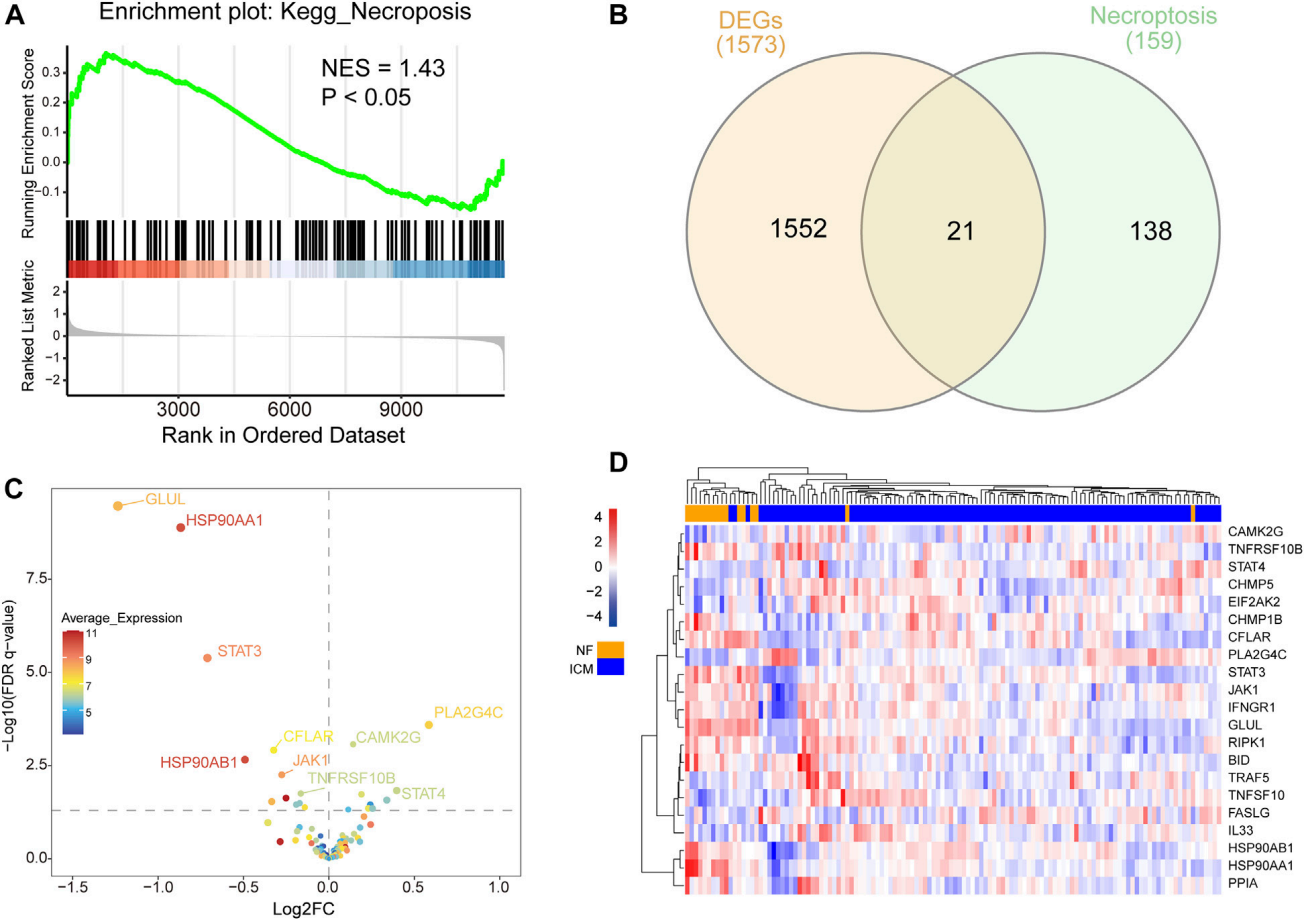
**(A)** Differential expression of necroptosis-related genes in heart samples of ICM and controls. **(B)** Venn diagram showing the overlap of genes between DEGs in GSE5406 and necroptosis-related genes in Kyoto Encyclopedia of Genes and Genomes pathway databases. **(C)** The volcano plot of NRDEGs. **(D)** Clustered heatmap of NRDEGs.

Gene set enrichment analysis (GSEA) was performed to identify predominant signaling pathways between ICM patients and non-failing (NF) controls. As shown in Figure 1A, the necroptosis pathway was considerably enriched and predominantly upregulated (normalized enrichment score = 1.43, *p* < 0.05), indicating an over strong link between necroptosis and ICM. A total of 1573 DEGs between ICM and NF samples were analyzed, with 703 genes up-regulated and 870 genes down-regulated. NRDEGs were identified based on DEGs and necroptosis gene set. We overlapped necroptosis pathway-related genes with DEGs in GSE5406. and obtained 21overlapping NRDEGs for further analysis (Figure 1B), of which 9 were up-regulated and 12 were down-regulated. The expression heatmap and volcano plot of NRDEGs are presented in (Figures 1C,D).

### 3.2 Construction of characteristic genes associated with progression and prognosis

**FIGURE 2:**
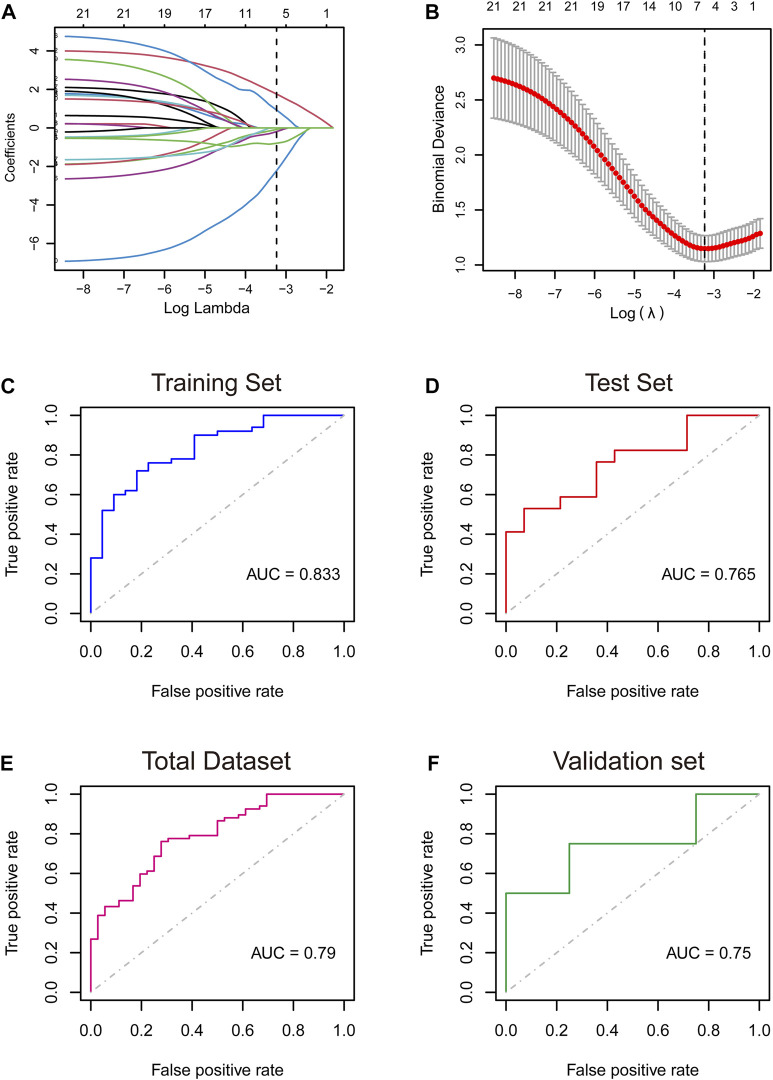
**(A,B)**: Least absolute shrinkage and selection operator (LASSO) logistic regression algorithm to screen key genes. **(C–F)**: Receiver operating characteristic (ROC) curves analysis of training set **(C)**, testing set **(D)**, total dataset **(E)**, and validation set **(F)**. AUC, area under the curve.

LVEF is a key factor in the progression and prognosis of ischemic cardiomyopathy. In patients with heart failure, reduced LVEF is strongly associated with adverse cardiac outcomes. Randomized clinical trials have shown that when patients with EF are severely reduced, below 15%, the mortality rate is greater than 50%, which is much higher than that of patients with LVEF of 16%–45%. Therefore, we used EF = 15% as the boundary to divide the patients into EF<=15% group (n = 67) and EF>15% groups (n = 36). ICM patients were randomly divided into a training cohort and a validation cohort at a ratio of 7:3. We applied the LASSO regression algorithm with an optimal lambda value of 0.0394 to select characteristic genes in NRDEGs in ICM patients (Figures 2A,B). Six genes, including *CFLAR*, *JAK1*, *STAT4*, *TNFSF10*, *CHMP1B*, and *CHMP5* with coefficients of 0.929, −0.795, 1.759, −0.080, −2.222, and −0.190, were identified to construct the NRDEGs-related progressive and prognostic signature. Higher scores represent more severe progression and worse prognosis of ICM patients. In training set, the AUC of the ROC curve was 0.833. In validation set, the AUC value of ROC curve was 0.765. In the overall set, the model achieved an ROC of 0.79 (Figures 2C–E). We did a validation using GSE203160 and found that the AUC reached 0.75 (Figure 2F), demonstrating that the NRDEGs-related signature possessed excellent diagnostic performance in predicting the progression of the progression and prognosis of ICM. In addition, we validated the expression of six NRS-related genes in ICM patients using two additional datasets (GSE57338 and GSE203160) and found that they were mostly differential expressed (Supplementary Figures S1, S2).

### 3.3 Genes regulatory network identifies transcription factor and miRNA associated with signature genes

**FIGURE 3:**
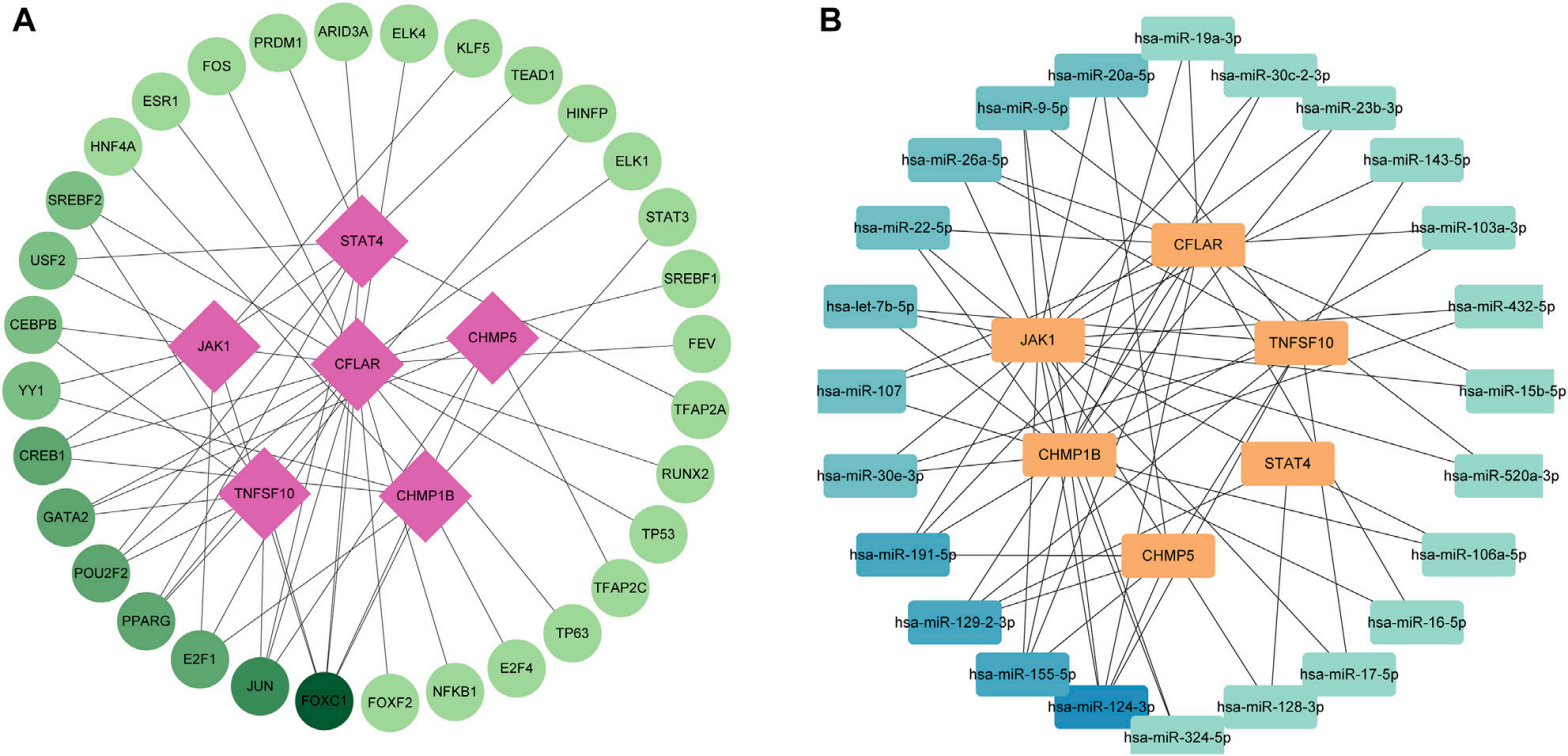
**(A)** Transcription factor-necroptosis-related differentially expressed genes (NRDEGs) regulatory network in ICM. The red squares represent NRDEGs, and the green dots represent transcription factors. The color of the edge lines in this network represents the correlation. **(B)** MiRNA-NRDEGs regulatory network in ICM. The yellow squares represent NRDEGs, and the blue squares represent miRNA. The color of the edge lines in this network represents the correlation.

The circles in the figure represent TFs, while the squares represent characteristic genes. The color of the nodes reflects the degree. Nodes with higher degrees are considered important hubs of the network. We found that *JUN* and *FOX1* are major transcription factors (Figure 3A). Meanwhile We identified a miRNA-NRDEGs’ interaction network, similar to previous analyses, we discover hsa-miR-20a-5p is a major regulator of signature genes (Figure 3B).

### 3.4 Identification of pathways associated with NRS

**FIGURE 4:**
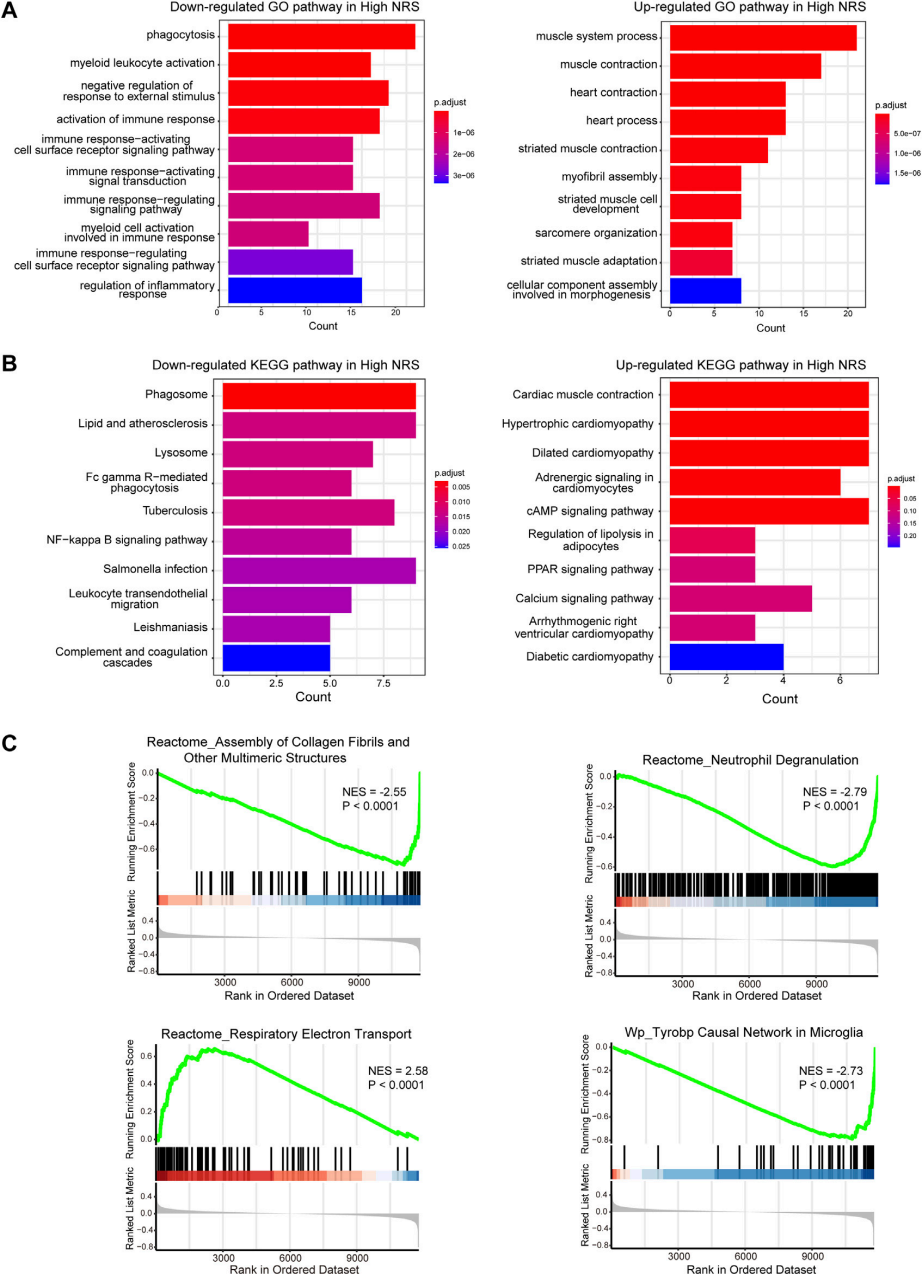
**(A)** GO functional analysis of genes in high NRS. **(B)** KEGG pathway analysis of genes in high NRS. **(C)** GSEA identifies signaling pathways involved in high NRS.

We identified 1573 DEGs associated with the necroptotic phenotype. The GO enrichment analysis revealed enrichment of phagocytosis, myeloid leukocyte activation, negative regulation of response to external stimulus, activation of immune response was downregulated in high-NRS group. Furthermore, enrichment of muscle system process, muscle contraction was found up regulated in high-NRS group (Figure 4A). Most importantly, the KEGG analysis revealed that DEGs tended to be enriched in the following terms: cardiac muscle contraction, hypertrophic cardiomyopathy (Figure 4B). GSEA analysis indicated assembly of collagen fibrils and other multimeric structures, neutrophil degranulation, Tyrobp causal network in microglia was down-regulated in high-NRS group. Meanwhile, respiratory electron transport was up -regulated in high-NRS group (Figure 4C).

### 3.5 Differences of immune infiltration in patients with high and low NRS

**FIGURE 5:**
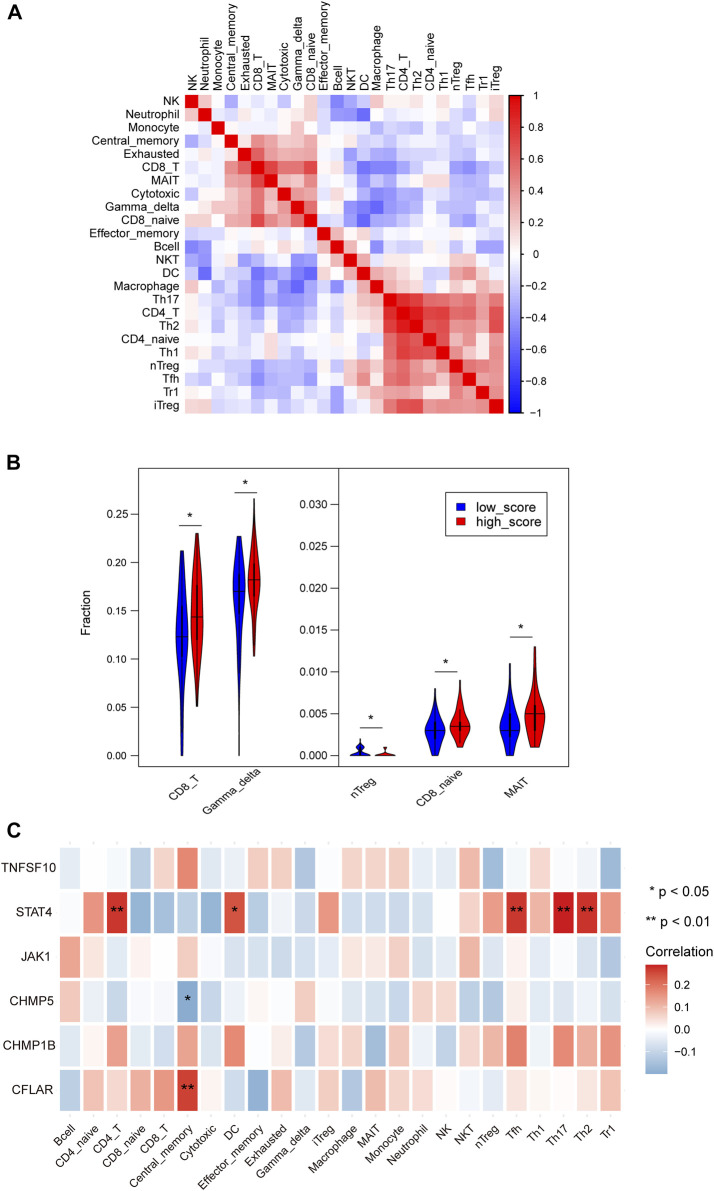
**(A)** Heatmaps depicting the correlations between distinct immune cell compositions. **(B)** The violin plot showed the statistically different immune infiltration score between the high-NRS and low-NRS groups in myocardium. **(C)** Correlation analysis of immune cell infiltrations with characteristic genes.

Among 24 immune cells, there were 5 cell types with significant differences in high-score groups compared with low-score groups. Among the 5 cell types, the score of nTreg cells in the low-score group was significantly enriched, whereas CD8-T cells, Gamma-delta cells, CD8-naive cells, mucosa-associated lymphocytes (MALT) were noticeably enriched in the high-score group (Figures 5A,B; Supplementary Figure S3). The STAT4 was predominantly favorably correlated with CD4-T cells, DC, Tfh, Th17, and Th2 cell infiltration and *CFLAR* was primarily positively correlated with central memory cell infiltration, meanwhile, *CHMP5* was negatively correlated with central memory cells (Figure 5C).

### 3.6 AZD-7762 as a potential therapeutic agent for patients with high NRS

**FIGURE 6:**
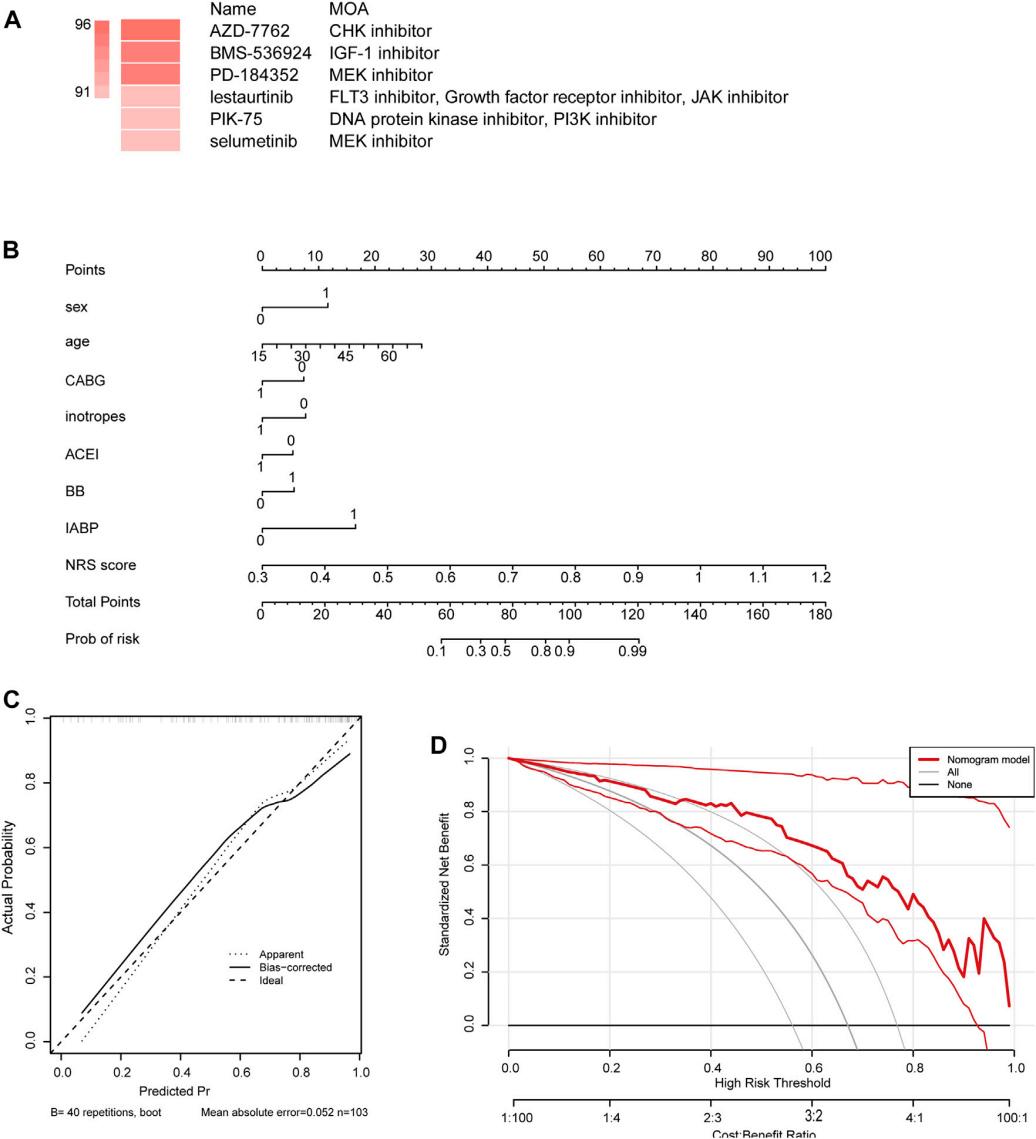
**(A)** Potential anti-ICM small molecule compounds were predicted by CMap analysis. **(B)** Establishment of a nomogram integrating characteristic genes for predicting ICM. In the nomogram, each variable corresponds to a score, and the total score can be calculated by adding the scores for all variables. **(C)** Calibration curve estimates the prediction accuracy of the nomogram. **(D)** Decision curve analysis shows the clinical benefit of the nomogram.

To find targeted drugs for high-risk patients, Potential anti-ICM small molecule compounds were predicted by CMap analysis, as shown in (Figure 6A). Drugs AZD-7762, BMS-536924, PD-1 84352 scored higher, they were CHK inhibitor, IGF inhibitor, MEK inhibitor. The results showed that they might have an intervening effect on ICM progression.

### 3.7 Establishment of a signature gene-based nomogram for ICM progression and prognosis

We constructed a nomogram as a diagnostic tool for ICM progression. In the nomogram, each factor, including sex, age, coronary artery bypass grafting (CABG), inotropes, angiotensin converting enzyme inhibitor (ACEI), beta-block (BB), intra-aortic balloon counter pulsation (IABP), NRS, corresponds to a score, and the total score is obtained by adding the scores of all characteristic genes (Figure 6B). The total score corresponds to the different risks of the ICM. The calibration curve showed that the nomogram was able to accurately estimate the progression of ICM (Figure 6C). Patients diagnosed with ICM can be benefit from a nomogram, as shown by decision curve analysis (Figure 6D).

### 3.8 Validation of the key genes in the heart tissues

**FIGURE 7:**
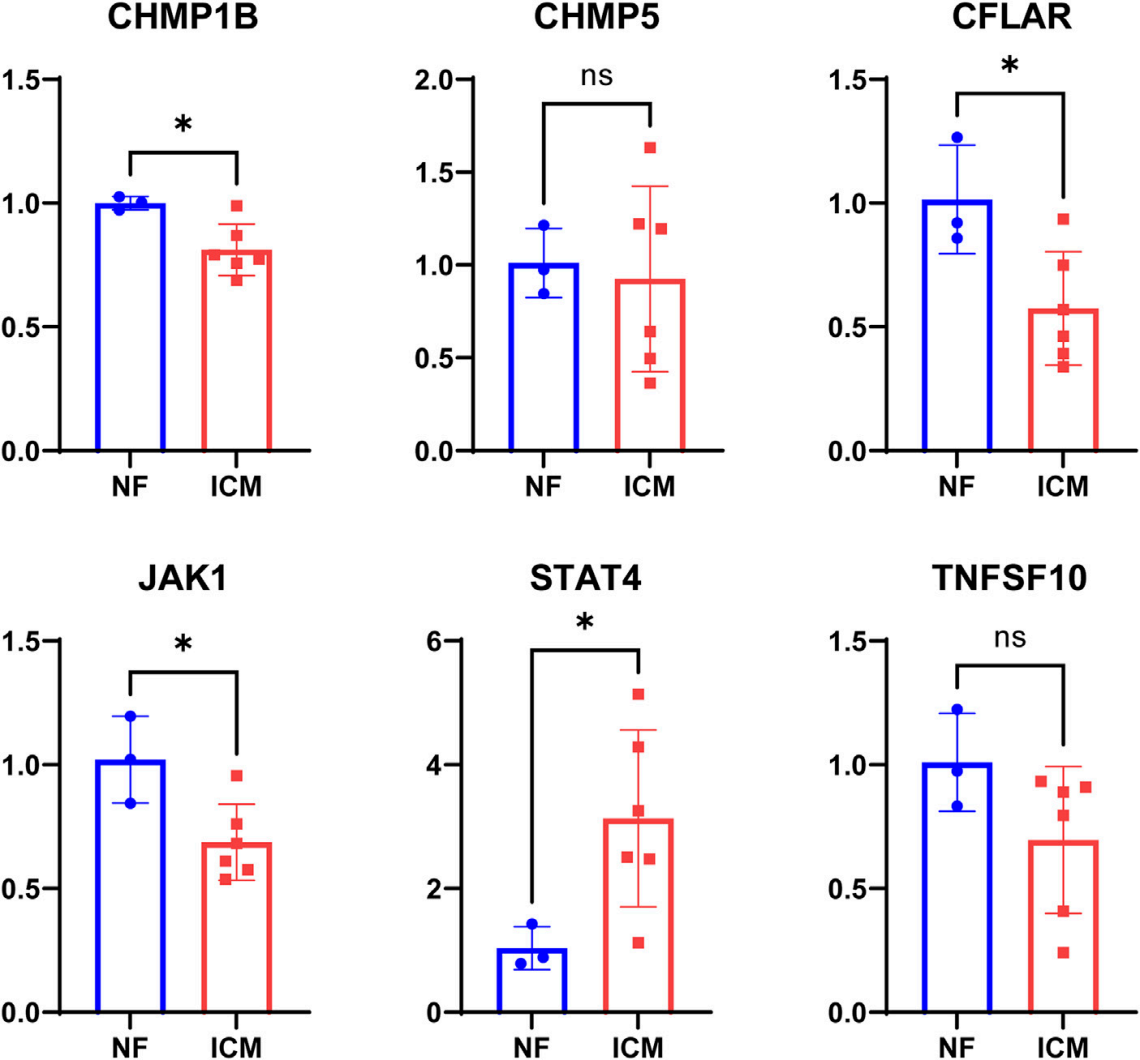
The levels of STAT4, TNFSF10, CHMP5, CHMP1B, JAK1, CFLAR mRNA expression in myocardium tissue from non-failing (NF) donor and ICM patients.

*STAT4* were expressed at a higher level and *CFLAR*, *CHMP1B*, *JAK1* were expressed at lower levels in the ICM heart tissues than in the NF heart tissues (*p* < 0.001), whereas *CHMP5*, *TNFSF10* showed no significant differences in expression level between the two groups (Figure 7).

## 4 Discussion

Ischemic heart disease remains the leading causes of morbidity and mortality and a major burden on worldwide healthcare (Severino et al., 2020). Recent studies have shown the pathological process of ischemic cardiomyopathy includes chronic myocardial ischemia and ischemia/reperfusion injury, which was closely associated with immune cell infiltration and necroptosis (Antman and Braunwald, 2020).

First, results from our analysis confirm previous research indicating that necroptosis pathway was considerably enriched and predominantly upregulated in ICM samples. Then, we overlapped necroptosis pathway-related genes with DEGs and obtained 21overlapping NRDEGs for further analysis. We applied the LASSO regression algorithm to select necroptosis-related characteristic genes in NRDEGs in ICM patients. Six genes, including *STAT4*, *TNFSF10*, *CHMP5*, *CHMP1B*, *JAK1*, and *CFLAR* were identified to construct the necroptosis-related progressive and prognostic signature. We found that among these genes, *CFLAR*, *CHMP1B*, *JAK1*, and *STAT4* were the most prominent necroptosis -related signature genes between the myocardial tissue of the ICM patients and those of the NF control.

Our results show that *CHMP1B*, *CHMP5*, *JAK1*, and *TNFSF10* have minus coefficients in the model, suggesting that their expression is opposite to the severity of the disease. Given that necroptosis is elevated in ICM patients, we hypothesized that they may inhibit the necroptotic pathway. CHMP1B and CHMP5, both belong to the chromatin-modifying protein/charged multivesicular body protein (CHMP) family, is required for both endocytic multivesicular bodies formation and regulation of cell cycle progression (Howard et al., 2001; Shim et al., 2006). It has been reported that in CHMP5-deficient glioma cells, both the granzyme B/perforin apoptotic pathway and the apoptosis-inducing factor-mediated necrosis pathway are activated, independently of the intrinsic and extrinsic apoptotic pathways (Shahmoradgoli et al., 2011; Wang et al., 2013). CHMP5 also promotes the stabilization of the intracellular pro-survival protein BCL2 in T cells and is rescued by the deletion of the pro-apoptotic protein BIM (Adoro et al., 2017). Similarly, the deletion of CHMP1 increased the susceptibility of renal tubular cells to death (Guan et al., 2021). This suggests that CHMP5 and CHMP1B may negatively regulate the necroptotic pathway in ICM patients. Previous studies on JAK1 have been focused mainly on the field of the proliferation and differentiation of tumor cells and some autoimmune disease. Sustained activation of signaling that inhibits JAK can lead to accelerated apoptosis of cells. In rheumatoid arthritis, continuous activation of JAK signaling in synovial joints results in the most pronounced “apoptosis resistance” in apoptotic chondrocytes (Malemud, 2018). Tofacitinib or ruxolitinib, JAK small molecule inhibitor, downregulated myeloid cell leukemia-1 mRNA level and decreased its protein level, which enabled BAK to trigger necroptosis (Thapa et al., 2013; Li et al., 2021). Therefore, we speculate that the JAK1 is associated with the negative regulation of necroptosis in ICM. Current research on TNFSF10 is insufficient. TNFSF10 is a protein functioning as a ligand that induces the process of cell death called apoptosis, but has also been implicated as a pathogenic or protective factor in various pulmonary diseases (Wiley et al., 1995). Given the minute coefficient of *TNFSF10* (0.08), we suggest that it may have a minor role in necroptosis-induced ICM progression. Our study showed that *STAT4* and *CFLAR* have positive coefficients in the model. Previous studies have shown that STAT4 plays a role in promoting apoptosis in hepatocellular carcinoma and ovarian granulosa cells (Jiang et al., 2020). A study in the vascular smooth muscle cell (VSMC) showed that the effect of STAT4 on VSMC apoptosis was mainly mediated by the activation of the mitochondrial apoptotic pathway (Lv et al., 2014). C-FLICE-like inhibitory protein (C-FLIP, CFLAR), a protein that not only inhibits apoptosis signaling but also modulates additional cell death pathways. Regulation of CFLAR expression represents a general tool to master cell death signaling pathways (Fulda, 2013). Therefore, STAT4 and CFLAR may contribute to the progression of ICM.

The identified GO category “myeloid leukocyte activation, negative regulation of response to external stimulus, activation of immune response” fits well with the concept emphasizing that Immunomodulatory is one of the most important factors predisposing to ventricular remodeling and coronary vascular occlusion in ICM (Geng, 2003; Abdel-Latif et al., 2015; El Bakry et al., 2017). The significantly enriched functions KEGG analysis were associated with cardiac muscle contraction, hypertrophic cardiomyopathy. The enriched GSEA pathways included the signaling pathways involved in inflammation, immunity, cell migration, and proliferation. These pathways included assembly of collagen fibrils and other multimetric structures, neutrophil degranulation, respiratory electron transport These findings suggest that these signaling pathways play a critical role in the ventricular and immune responses in ICM.

Recent studies have shown that in the pathogenesis of chronic ischemic heart failure exposure of autoantigens in ischemic necrotic myocardial tissue activates immune cells and aggravates tissue damage and ventricular remodeling (Bansal et al., 2017). Hence, ICM is considered a chronic state of aberrant immune activation and inflammatory response in this study. We found that nTreg cells in the low-score group were significantly enriched, whereas CD8-T cells, Gamma-delta cells, CD8-naive cells, mucosa-associated lymphocytes (MALT) were noticeably enriched in the high-score group. After acute MI, Treg cells delay ventricular remodeling by regulating macrophage phenotype, suppressing inflammatory responses, and promoting ventricular muscle repair (Bansal et al., 2019). Our results showed that STAT4 was positively correlated with Tfh, Th17, and Th2. However, their abundance values were close to zero, and we believe that these results may have relatively little practical significance.

Our analysis shows that ICM are widely involved in a series of pathological processes such as necroptosis, immunity, inflammation and cardiac fibrosis. Myeloid leukocyte activation, immune response and NF-kappa B signaling were down-regulated in high NRS, but cardiac muscle contraction and hypertrophic cardiomyopathy was up-regulated in high NRS. Meanwhile There was a few immune cell infiltrates showing significantly difference between the high NRS group and low NRS group. We speculated necroptosis may be more involved in myocardial fibrosis than inflammation.

AZD-7762 (AZD) (S)-55-(3-Fluorophenyl)-N-(piperidin-3-yl)-3-ureidothiophene-2-carboxamide is a small molecule inhibitor of WEE1 protein kinase. WEE1 controls the program of entry into mitosis after DNA damage, inhibiting the progression of cells from G2 to M phase, giving cells ample time to repair their damaged. Research on the role of WEEI kinase has mainly focused on the field of cancer and is limited in ICM. The current study provides evidence that damaged cells can activate WEE1 kinase, which prevents apoptosis and cell death by inducing cell cycle arrest at G2 phase. Therefore, inhibition of WEE1 kinase could sensitize cancer cells to chemotherapeutic drugs. Therefore, WEEI kinase may be related to myocardial apoptosis and myocardial repair after myocardial infarction (Lv et al., 2014). Moreover, as depicted in our research curve analysis, the patients diagnosed with ischemic heart disease could clinically benefit from the nomogram.

## 5 Conclusion

In conclusion, we have described characteristic genes associated with necroptosis as a useful and non-invasive tool for LVEF classification in ICM patients, and identified pathways, immune cells and targeted drugs in high-risk patient groups. This is the first necroptosis-related feature in ICM that can evaluate progression and prognosis in ICM patients. However, some important limitations remain in our study. The sample size included in this study was insufficient, and there was an imbalance in the number of patients in both groups, which may affect the accuracy of the model. Larger cohorts are needed to validate the robustness and utility of the prognostic model in future clinical practice. In addition, more experiments will be performed to further elucidate the role of signature genes in ICM.

## Data Availability

The datasets presented in this study can be found in online repositories. The names of the repository/repositories and accession number(s) can be found in the article/Supplementary Material.
