# Supplementary material for: A risk model developed based on necroptosis to assess progression for ischemic cardiomyopathy and identify possible therapeutic drugs
Source: Front Pharmacol. 2022 Nov 28;13:1039857. doi: 10.3389/fphar.2022.1039857 (PMC9744324; doi:10.3389/fphar.2022.1039857)
Supplement: Supplementary file 1 [file DataSheet1.docx]

Supplementary Material

# Supplementary Figures 1-3

# Supplementary Table 1


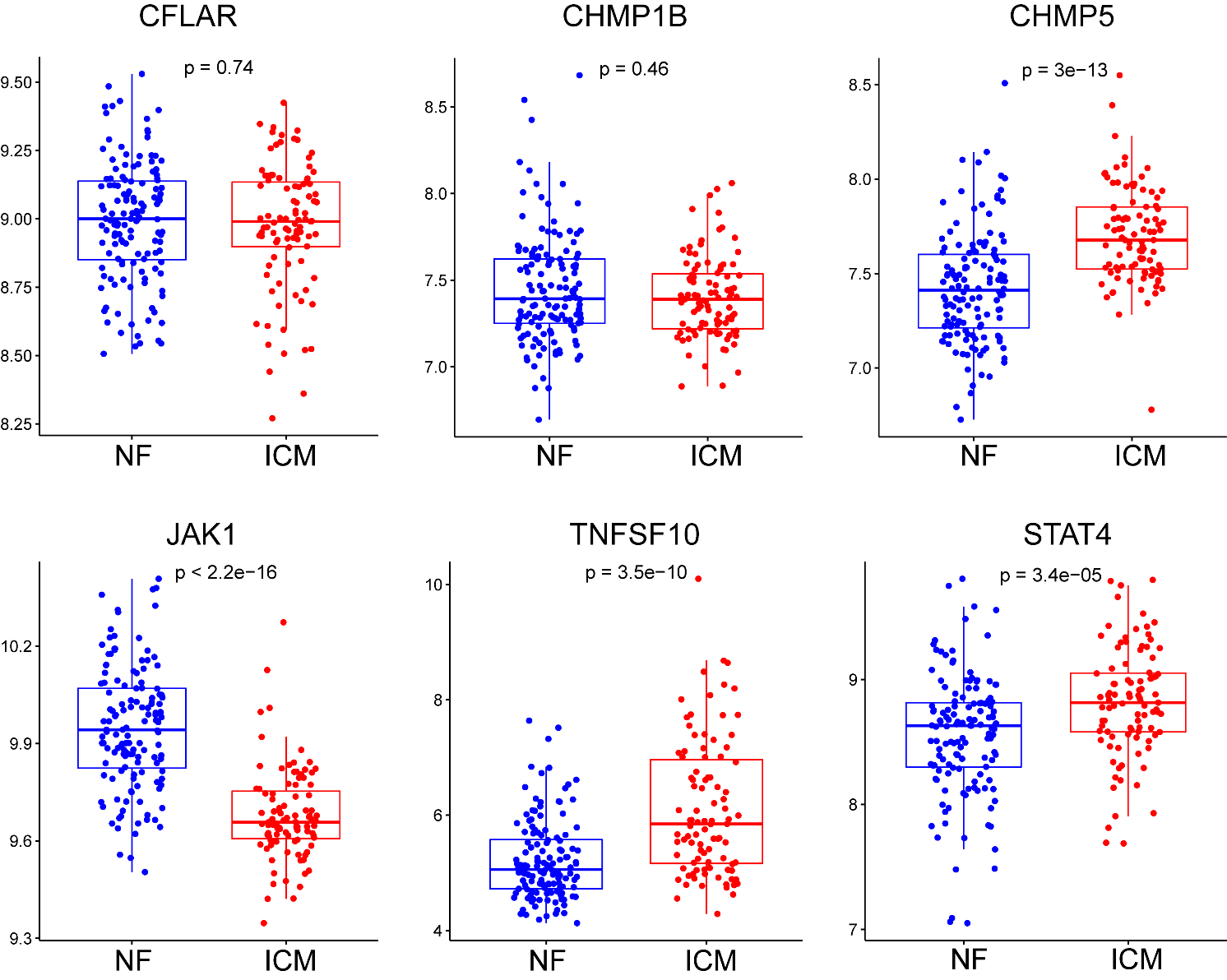


**Supplementary Figure 1.** The levels of CFLAR, CHMP1B, CHMP5, JAK1, STAT4 expression in myocardium tissue from non-heart failure and ICM patients in GSE57338.


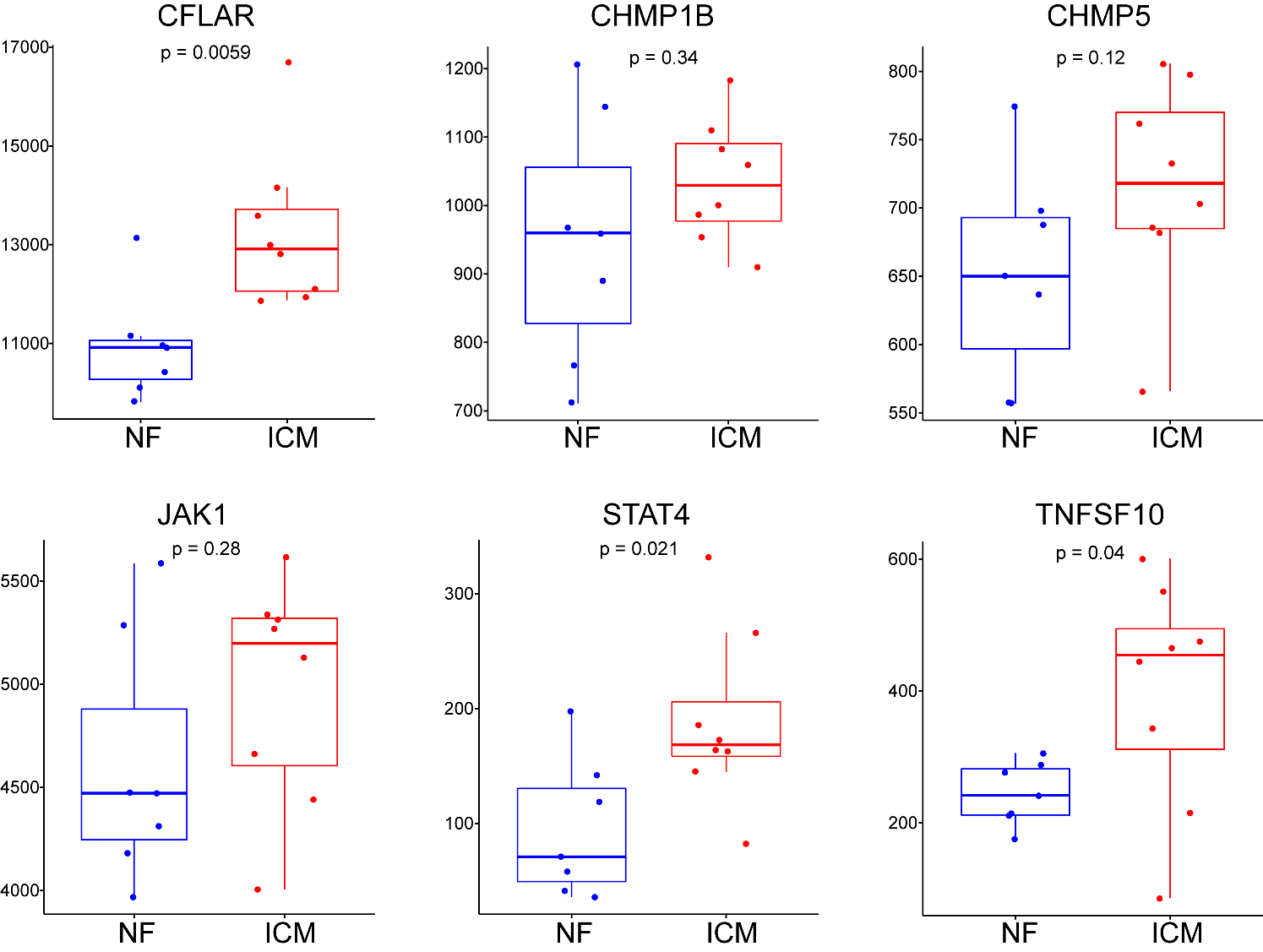


**Supplementary Figure 2.** The levels of CFLAR, CHMP1B, CHMP5, JAK1, STAT4 expression in myocardium tissue from non-heart failure and ICM patients in GSE203160.

**Supplementary Figure 3.** A and B**:** The violin plot showed the statistically different immune infiltration score between the high-NRS and low-NRS groups in myocardium.
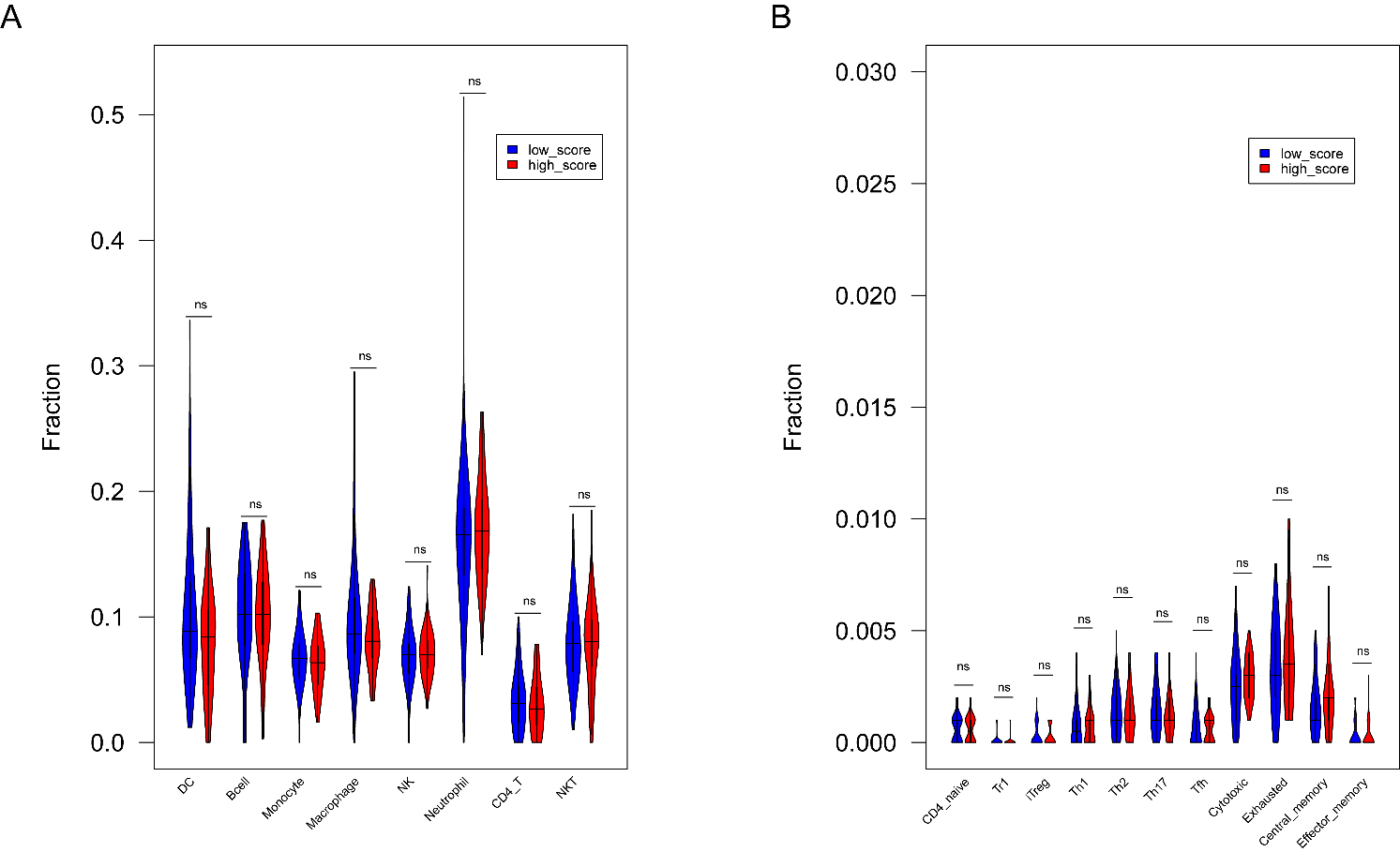


|  | Male | Age | NYHA classification | LVEDD (cm) | LVFS (%) | LVEF (%) | NT-proBNP (pg/ml) |
| --- | --- | --- | --- | --- | --- | --- | --- |
| ICM1 | + | 52 | IV | 6.4 | 13 | 22.5 | 3339 |
| ICM2 | - | 30 | IV | 6.8 | 10 | 27 | 9481 |
| ICM3 | + | 47 | IV | 8.2 | 8 | 12.6 | 3290 |
| ICM4 | + | 46 | IV | 6.8 | 7 | 15.9 | 1161 |
| ICM5 | + | 62 | IV | 7.7 | 6 | 11.5 | 2450 |
| ICM6 | + | 41 | IV | 6.6 | 19 | 38 | 491 |

**Supplementary Figure Table 1**. Clinical characteristics of 6 ICM patients.

LDEDD: Left ventricular end-diastolic diameter. LVFS: Left ventricular fraction shortening. LVEF: Left ventricular ejection fraction. NT-proBNP: N-terminal-pro hormone BNP.
